# A minimal cross-kingdom SynCom promotes plant growth and suppresses wheat crown rot via coordinated rhizosphere microbiome remodeling

**DOI:** 10.3389/fpls.2026.1758273

**Published:** 2026-02-25

**Authors:** Qian Zhou, Yuzhou Wang, Tingting Zhou, Kaidiriye Yusupu, Dan Gao, Huixin Zhao, Liufeng Ma

**Affiliations:** 1 College of Life and Geography Sciences, Key Laboratory of Biological Resources and Ecology of Pamirs Plateau in Xinjiang Uygur Autonomous Region, College of Modern Agriculture, The Center for Biotechnology Breeding and Intelligent Cultivation of Crops in South Xinjiang, Kashi University, Kashi, China; 2 Xinjiang Key Laboratory of Special Species Conservation and Regulatory Biology, School of Life Sciences, Xinjiang Normal University, Urumqi, China; 3 Institute of Plant Protection, Xinjiang Uygur Autonomous Region Academy of Agricultural Sciences, Urumqi, China; 4 Institute of Chinese Materia Medica, China Academy of Chinese Medical Sciences, Beijing, China

**Keywords:** cross-kingdom consortium, plant growth promotion, rhizosphere microbiome, synthetic microbial community, wheat crown rot

## Abstract

**Introduction:**

Wheat crown rot (WCR) caused by Fusarium pseudograminearum threatens wheat productivity, and sustainable control strategies are urgently needed.

**Methods:**

We constructed a minimal cross-kingdom synthetic community (SynCom) consisting of Trichoderma harzianum T19 and Bacillus rugosus PM16, and evaluated its effects on wheat growth and WCR suppression. Rhizosphere microbiome assembly (full-length 16S rRNA/ITS sequencing) and metabolomic shifts were assessed to elucidate mechanisms.

**Results:**

The SynCom significantly suppressed WCR and promoted wheat growth under pathogen pressure, improving biomass, chlorophyll content, and yield-related traits. SynCom inoculation remodeled the rhizosphere microbiome by enriching beneficial taxa (e.g., Mortierella) and reducing pathogen-associated fungi, and it enhanced rhizosphere enzyme activities and nutrient availability. Metabolomics revealed accumulation of growth-promoting and defense-related metabolites, supporting coordinated microbiome–metabolome regulation.

**Discussion:**

A minimal cross-kingdom SynCom can establish a disease-suppressive and growth-promoting rhizosphere through coordinated restructuring of microbial communities and metabolites, highlighting its potential as an eco-friendly strategy for WCR management.

## 1 Introduction

Wheat crown rot (WCR) is a soil-borne disease that poses a significant threat to wheat production, primarily caused by pathogens such as *Fusarium pseudograminearum*, *Fusarium graminearum*, and other *Fusarium* species (Ma et al., 2024; Xie et al., 2024). The disease primarily infects the wheat crown, disrupting the plant’s water and nutrient transport systems, leading to browning and gradual rotting of the crown. This severely affects the plant’s health and yield. During the grain filling stage, the infection often leads to “whiteheads,” where the heads fail to fully fill with grain, further exacerbating yield loss (Wang et al., 2023; Li et al., 2022).

WCR is widespread globally, especially in countries such as Australia, South Africa, the United States, and Canada, where it causes extensive damage (Kazan and Gardiner, 2018). In typical years, WCR can reduce wheat yield by about 9.5%; in years of severe outbreaks, yield losses can reach as high as 35% (Zhang et al., 2015). In China, WCR caused by *F. pseudograminearum* was first reported in 2012, and the disease has since been widely reported across major winter wheat-growing regions (Li et al., 2012). In recent years, factors such as excessive fertilization, improper straw management, and poor disease resistance in wheat varieties have led to an increasing and spreading incidence of WCR in the Huang-Huai wheat production area. This trend makes the control of WCR an urgent issue. WCR not only affects the growth and development of wheat but also impacts grain filling, reducing both yield and quality, which in turn affects food security. Traditional chemical control methods, such as the use of chemical pesticides and soil fumigation, can mitigate the disease in the short term. However, these methods face sustainability challenges due to their negative environmental impact and the increasing resistance of pathogens. Therefore, it is crucial to explore new, more environmentally friendly control strategies.

In recent years, microbial biocontrol has emerged as an alternative to traditional chemical pesticides and has gained significant attention. The plant-associated microbiome, particularly the rhizosphere microbiota, plays a vital role in plant health, growth, and disease resistance (Ketehouli et al., 2024). The rhizosphere microbiota forms a complex ecological network with plant roots, which can effectively suppress pathogenic microorganisms, regulate plant immune responses, and promote plant growth. Among the microbial biocontrol strategies, synthetic microbial communities (SynComs) have gained attention as an innovative approach. By combining different beneficial microorganisms, SynComs mimic and reconstruct the natural ecosystem of plant rhizospheres, improving microbial community diversity and stability, thereby enhancing plant disease resistance.

SynComs offer significant advantages over single microbial strains. First, SynComs can better mimic the complex ecological environment of the rhizosphere and promote synergistic interactions between microorganisms to enhance disease resistance. For example, bacteria such as *Bacillus*, *Pseudomonas*, and *Streptomyces* play a crucial role in regulating the rhizosphere microbial community, promoting plant growth, and enhancing disease resistance through the secretion of antimicrobial agents, plant hormones, and nutrients (Liu et al., 2024a). Additionally, SynComs show superior environmental adaptability. Studies have shown that SynComs can stabilize and grow in harsh soil environments, enhancing plant immunity and suppressing pathogen growth through various mechanisms (Zhang, 2024).

For instance, a study by Zhou et al. (2022a) demonstrated that cross-kingdom SynComs (including both bacteria and fungi) were more effective in suppressing *Fusarium* wilt in tomatoes than bacterial or fungal communities alone. This study indicated that the combined advantages of bacteria and fungi in cross-kingdom communities allowed for more effective disease suppression through various mechanisms, such as antimicrobial secretion, competitive exclusion of pathogens, and boosting plant immunity (Zhang et al., 2025). Similar studies in wheat have shown that SynComs can effectively control WCR and enhance plant resistance (Yin et al., 2022; Wang et al., 2025b).

This study evaluates a minimal cross-kingdom SynCom for its ability to modulate the wheat rhizosphere microbiome and suppress WCR. By constructing an optimal minimal SynCom, we analyze its role in improving the structure of wheat root-associated microbial communities, promoting wheat growth, and enhancing disease resistance, providing new approaches for the green control of WCR.

## 2 Materials and methods

### 2.1 Study location

The pot experiment was conducted in the tissue culture chamber at Xinjiang Normal University (Urumqi, Xinjiang, China) under controlled conditions (25°C, 16 h light/8 h dark).

### 2.2 Strain origin and identification

PM16 and T19 were isolated from rhizosphere soil of healthy wheat plants collected from the experimental field of Xinjiang Normal University (Urumqi, Xinjiang, China). PM16 was identified as *Bacillus rugosus* based on 16S rRNA gene sequencing (27F/1492R), and T19 was identified as *Trichoderma harzianum* based on ITS sequencing (ITS1F/LR3) (NCBI BLAST; ≥99% identity). Both strains are maintained at Xinjiang Normal University. The *F. pseudograminearum* isolate Fp-XJ-07 was obtained from symptomatic wheat crown tissues collected in Xinjiang and confirmed by morphology and ITS sequence analysis.

### 2.3 Plant growth–promoting trait assays

IAA production by PM16 was quantified using the modified colorimetric method of Sarwar and Kremer. Phosphate solubilization was assessed using NBRIP medium (Chinook, CN260728) with tricalcium phosphate [Ca_3_(PO_4_)_2,_ 5.0 g/L] as the insoluble inorganic phosphate source. Siderophore production was determined using the Chrome Azurol S (CAS) assay (Kumar et al., 2018).

### 2.4 Antagonism and compatibility assays

The antagonistic activity of T19 against Fp was evaluated using dual-culture assays on PDA and TSA media. Antagonism was quantified as percent growth inhibition calculated as (Rc−Rt)/Rc × 100, where Rc and Rt represent radial growth of Fp in control and dual-culture plates, respectively. For compatibility assays, 2 μL of PM16 suspension was inoculated on two opposite sides of PDA plates, and a 6-mm plug of T19 was placed at the center. Plates were incubated at 28 °C, and the presence/absence of visible inhibition zones was recorded (Qiao et al., 2024).

### 2.5 Inoculum preparation and construction of the minimal cross-kingdom SynCom

The conidia of *Trichoderma* strain T19 were added to carboxymethyl cellulose liquid medium and incubated at 28°C for 5 days. The spore suspension was then adjusted to a final concentration of 10^6^ spores/mL using sterile water. Strain PM16 was cultured in nutrient broth at 37°C for 8 h, and the cell density was adjusted to OD_600_ = 0.1, followed by resuspension in sterile water. Equal volumes (v/v) of the T19 spore suspension and PM16 bacterial suspension were mixed to construct a two-member minimal cross-kingdom synthetic community (SynCom, TB) (Shao et al., 2025; Zhu et al., 2024).

### 2.6 Pot experiment for biocontrol efficacy against WCR

Wheat (*Triticum aestivum* L., cv. Hechun 213) was subjected to four treatments: a sterile-water control (CK), inoculation with *F. pseudograminearum* (Fp), inoculation with the minimal cross-kingdom SynCom (TB), and co-inoculation with Fp and TB (TB-Fp). The pot experiment included six independent biological replicates per treatment, with ten plants per replicate, and was arranged in a randomized complete block design. Each pot was filled with 1100 g of dried, non-sterile topsoil that had been air-dried and passed through a 2-mm sieve before use. Prior to pot establishment, a composite subsample of this bulk soil was analyzed for baseline physicochemical properties using standard protocols (De Vries et al., 2018); the initial values are summarized in **Supplementary Table S1**. Fp inoculation was performed on Day 0 by soil drenching each pot with 200 mL of a conidial suspension (10^6^ spores/mL). TB was applied on Day 7 using the same soil-drenching method (200 mL per pot). TB was freshly prepared immediately before application by mixing equal volumes (1:1, v/v) of the T19 spore suspension (10^6^ spores/mL) and PM16 suspension (OD_600_ = 0.1). Disease assessment was performed 45 days after pathogen inoculation. Disease severity was visually rated for each plant at 45 days post-inoculation using a 0−9 scale (Yang et al., 2019), where higher scores indicate more severe crown rot symptoms. The disease index (DI, %) was calculated as: DI (%) = [Σ(r_i_ × n_i_)/(R × N)] × 100, where r_i_ is the disease rating (0-9) for severity class *i*, n_i_ is the number of plants in class *i*, R is the maximum rating (9), and N is the total number of assessed plants. For each inoculation treatment, three rhizosphere soil samples were randomly collected. These samples were subjected to high-throughput sequencing of 16S rRNA and ITS regions, as well as soil metabolomic profiling.

### 2.7 Measurement of wheat physiological traits, rhizosphere soil properties, and enzyme activities

Wheat growth parameters, including plant height, fresh and dry weight of shoots and roots, chlorophyll content, root length, and stem diameter, were measured as described by Ghanem et al. (2025). Thousand-kernel weight (TKW) was determined after plant maturation, with three replicates per treatment and 20 spikes per replicate (Zhang et al., 2021). Physiological enzyme activities in wheat leaves were quantified using commercial assay kits (Suzhou Geruisi Biotechnology Co., Ltd., China) following the manufacturer’s instructions (Guo et al., 2024).

After 45 days of inoculation with the minimal SynCom, rhizosphere soil samples were collected from potted wheat plants. Soil enzyme activities, including acid phosphatase, urease, sucrase, and catalase, were determined using commercial assay kits (Suzhou Geruisi Biotechnology Co., Ltd., China) according to the manufacturer’s instructions. Rhizosphere soil physicochemical properties, including pH, electrical conductivity (EC), soil organic matter (SOM), total nitrogen (TN), total phosphorus (TP), total potassium (TK), available nitrogen (AN), available phosphorus (AP), and available potassium (AK), were measured using standard protocols (De Vries et al., 2018).

### 2.8 Full-length 16S rRNA/ITS amplicon sequencing and bioinformatic processing

Total DNA was extracted from 500 mg rhizosphere soil using a commercial soil DNA extraction kit following the manufacturer’s protocol. For bacterial community profiling, the full-length 16S rRNA gene (~1,500 bp; covering V1–V9) was amplified using primers 27F (5′-barcode+AGAGTTTGATCMTGGCTCAG-3′) and 1492R (5′-ACCTTGTTACGACTT-3′). For fungal community profiling, the full-length ITS region (~1,300 bp) was amplified using primers ITS1F (5′-barcode+CTTGGTCATTTAGAGGAAGTAA-3′) and LR3 (5′-CCGTGTTTCAAGACGGG-3′). PCR was performed using a high-fidelity DNA polymerase under the following conditions: initial denaturation at 98°C for 5 min, followed by 25–30 cycles of 98°C for 30 s, 56°C for 30 s, and 72°C for 45 s, with a final extension at 72°C for 10 min. Amplicons were verified on 2% agarose gels, excised, and purified prior to library preparation.

Purified amplicons were used to construct third-generation sequencing libraries using the SMRTbell^®^ Express Template Prep Kit 3.0 (Pacific Biosciences). Libraries were quantified using the Quant-iT™ PicoGreen^®^ dsDNA Assay Kit, quality-checked, and sequenced on the PacBio Revio platform in Circular Consensus Sequencing (CCS; HiFi) mode.

Microbiome data were processed in QIIME 2 (v2024.5) following standard workflows recommended in the QIIME 2 documentation. Briefly, reads were demultiplexed using the demux plugin, and quality filtering, denoising, and chimera removal were performed with DADA2 to generate amplicon sequence variants (ASVs) and the corresponding feature table. Taxonomic assignment was performed in QIIME 2 using the q2-feature-classifier plugin by aligning representative ASV sequences against reference databases, using the NCBI database for full-length bacterial 16S rRNA sequences and the UNITE v9 database for full-length fungal ITS sequences. Because PNA clamps were not used, ASVs annotated as chloroplast or mitochondria were removed by taxonomy-based filtering prior to downstream diversity and community analyses. The resulting ASV table was used for subsequent statistical analyses.

### 2.9 Soil metabolomics analysis

Soil metabolites were extracted from 100 mg rhizosphere soil with 300 μL pre-cooled methanol containing 2-chlorophenylalanine (5 ppm; internal standard). Samples were bead-beaten (55 Hz, 60 s; twice), ultrasonicated for 10 min, incubated at −20°C for 30 min, and centrifuged (12,000 rpm, 10 min, 4°C). Supernatants were filtered (0.22 μm) and transferred to LC–MS vials. QC samples were prepared by pooling 10–20 μL from each extract; 2–4 QC injections were used for system conditioning, and QC was injected every 5–10 samples.

LC–MS analysis was performed on a Vanquish Flex UHPLC coupled to an Orbitrap Exploris 120 (Thermo) using an ACQUITY UPLC HSS T3 column (100 Å, 1.8 μm, 2.1 × 100 mm) at 40°C (flow rate 0.4 mL/min; injection volume 2 μL; autosampler 8°C). Mobile phase A was 0.1% formic acid in water and mobile phase B was 0.1% formic acid in acetonitrile. Data were acquired in positive and negative ion modes (HESI; spray voltage 3.5/−3.0 kV) with full-scan MS (m/z 70–1000; resolution 60,000) and DDA-MS/MS (Top 4; dynamic exclusion 4 s; MS/MS resolution 15,000; HCD 30%).

Raw data were processed using MS-DIAL (v4.9.221218). Features not detected in >50% of QC injections were removed, missing values were gap-filled, and the resulting relative abundance matrix was used for downstream analyses.

### 2.10 Microbial co-occurrence network construction

Co-occurrence networks were constructed using the absolute abundance of ASVs present in at least three replicate samples, with relative abundance among the top 500. These networks were constructed using the Spearman correlation matrix with the “ggClusterNet” package (Wen et al., 2025). Key network metrics were calculated, including nodes, edges, average path length, diameter, degree distribution, clustering coefficient, and modularity. Microbial taxa were classified as ecological units based on within-module connectivity (Zi) and among-module connectivity (Pi).

### 2.11 Statistical analysis

Statistical analyses were conducted using SPSS v.25.0, R v.4.4.1, and Python v.3.10.7. Microbiome data were processed using QIIME 2, including demultiplexing, quality filtering, and taxonomic assignment. Alpha diversity was primarily evaluated using Chao1 and Shannon indices as complementary measures of within-sample diversity: Chao1 reflects estimated richness, whereas Shannon captures both richness and evenness. β-diversity was assessed using Jaccard and Bray–Curtis dissimilarity analyses, with results visualized using Hierarchical Clustering Analysis (HCA) and Non-Metric Multidimensional Scaling (NMDS). Permutational multivariate analysis of variance (PERMANOVA) was used to test the effects of experimental treatments (CK, Fp, TB, and TB-Fp) on microbial community structure. Linear discriminant analysis effect size (LEfSe) was used to identify taxa with different characteristics. To identify microbial features that best discriminate treatments, Random Forest (RF) classification was performed using the ASV abundance table as input. Low-prevalence ASVs were removed to reduce sparsity prior to modeling. RF models were trained with treatment group as the response variable, and performance was assessed using out-of-bag (OOB) error and k-fold cross-validation. Feature importance was computed using standard RF importance measures, and the top-ranked discriminant ASVs were reported. This approach follows established RF applications in microbiome-related classification and biomarker screening (Zhou et al., 2022b, Zhou et al., 2024). ocurrence networks and associated clustering heatmaps were constructed to explore the relationship between DAMs (Differentially Abundant Metabolites) and microbial communities. Specific bioinformatics packages used in each analysis are detailed in the supplementary methods. Statistical significance was set at α = 0.05, with results presented as mean ± SD.

## 3 Results

### 3.1 Selection and identification of core strains in the minimal SynCom

From a wheat rhizosphere isolate collection, T19 and PM16 were selected as the core strains based on their antagonism against Fp and plant growth–promoting traits, respectively (**Supplementary Table S2**). Among the screened isolates, PM16 showed the strongest overall performance, with the largest phosphate-solubilization halo (1.95 ± 0.05 cm), the highest IAA production (48.67 ± 1.15 mg/L), and the largest siderophore halo (26.17 ± 1.04 mm) (**Supplementary Table S2**). T19 was selected as the fungal member based on its antagonistic activity against Fp, showing a mycelial inhibition rate of 55.76% in dual-culture assays.

**Figure 1:**
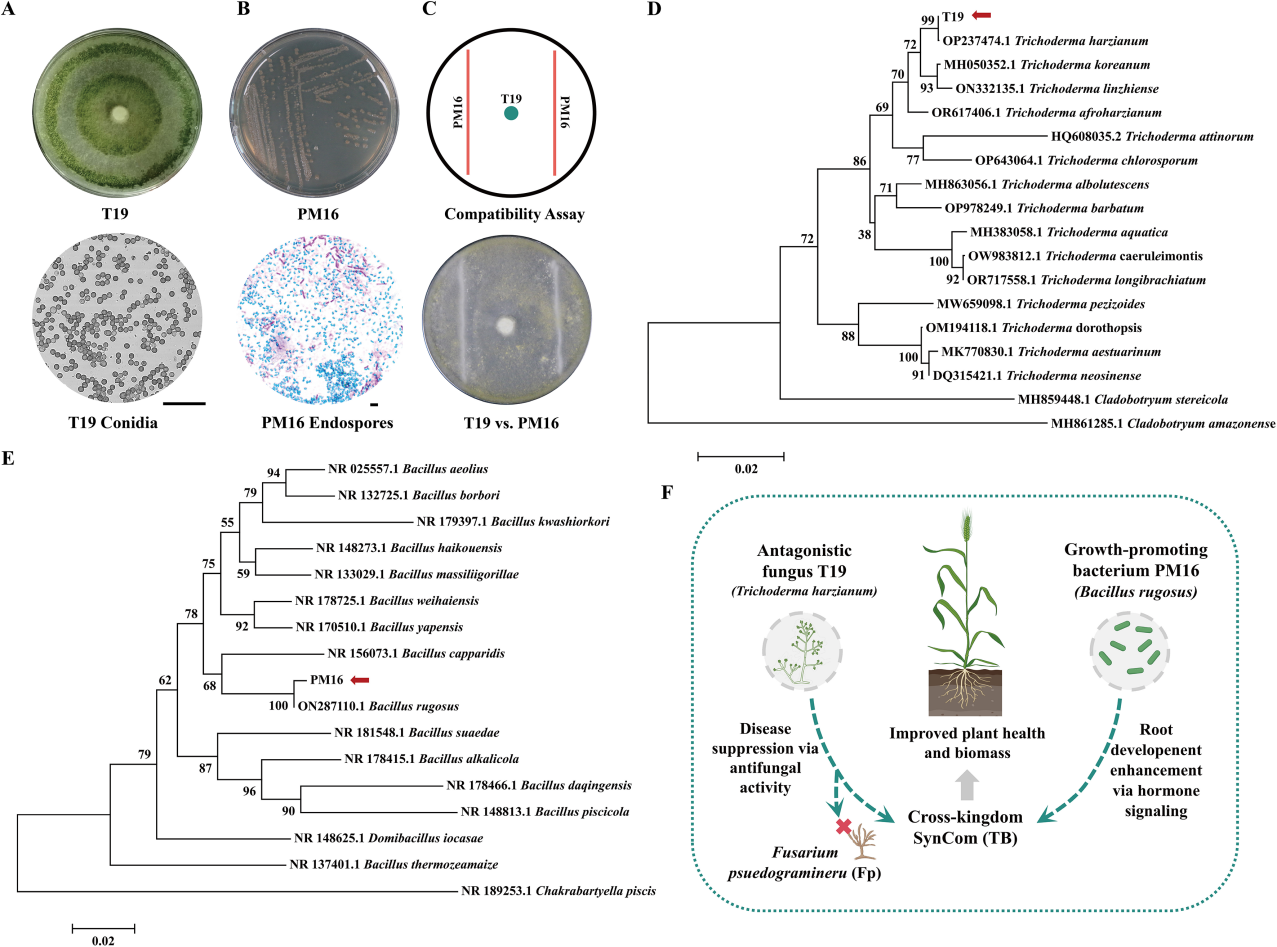
Identification and interaction mechanism analysis of the antagonistic fungus T19 and the growth-promoting bacterium PM16. **(A)** Colony morphology and conidial microstructure of T19. **(B)** Colony morphology of PM16 and spore staining. **(C)** Compatibility assay between T19 and PM16. **(D)** Phylogenetic tree of T19. **(E)** Phylogenetic tree of PM16. **(F)** Proposed mechanistic model of the cross-kingdom minimal SynCom TB constructed from T19 and PM16 in wheat.

Co-cultivation assays further indicated that T19 and PM16 were compatible, with no visible inhibition zones or antagonistic reactions observed on the same plate, supporting their stable coexistence and the feasibility of constructing a two-member minimal SynCom (**Figure 1C**). ITS-based phylogenetic analysis clustered T19 with *T. harzianum* (99% identity; **Figure 1D**), and 16S rRNA analysis clustered PM16 with *B. rugosus* (100% identity; **Figure 1E**). Together, morphological and molecular analyses confirmed the identities of T19 and PM16. Based on their complementary functional traits and compatibility, we assembled the two-member minimal SynCom TB to integrate growth promotion and disease suppression (**Figure 1F**). TB was selected through trait-based prioritization and compatibility testing, rather than via exhaustive combinatorial optimization across all possible isolate pairings.

### 3.2 Effects of the minimal SynCom TB on wheat growth and disease resistance

**Figure 2:**
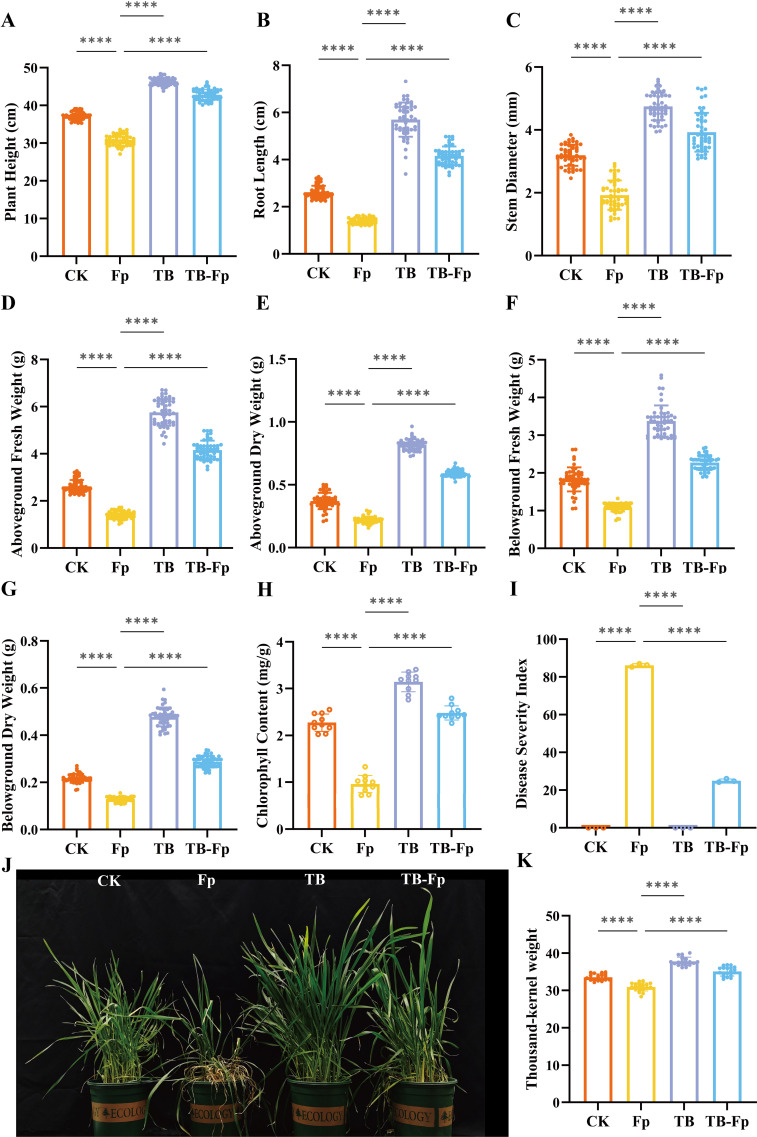
Effects of the minimal SynCom (TB) on wheat growth, disease resistance, and yield traits. Treatments included control (CK), pathogen inoculation (Fp), minimal SynCom inoculation (TB), and combined inoculation (TB-Fp). **(A)** Plant height; **(B)** Root length; **(C)** Stem diameter; **(D)** Aboveground fresh weight; **(E)** Aboveground dry weight; **(F)** Belowground fresh weight; **(G)** Belowground dry weight; **(H)** Chlorophyll content; **(I)** Disease severity index; **(J)** Representative phenotypes of wheat plants; **(K)** Thousand-kernel weight (TKW). Significance levels: ns, not significant; ****P < 0.0001.

TB treatment significantly enhanced wheat growth compared to the blank control (CK). Specifically, plant height, root length, and stem diameter increased by 24.63%, 117.69%, and 48.67%, respectively (**Figures 2A–C**). Aboveground fresh and dry biomass increased by 119.98% and 120.40%, while belowground fresh and dry biomass increased by 84.51% and 120.34%, respectively. Chlorophyll content was also elevated by 38.17% (**Figures 2D–H**). Under Fp-induced stress, TB significantly reduced the disease index, with a biocontrol efficacy of 70.97% (**Figure 2L**). Phenotypic observations revealed that TB-treated plants exhibited robust growth and darker green leaves, whereas Fp-infected plants showed evident wilting and chlorosis (**Figure 2J**). Even under pathogen stress, plants in the TB-Fp treatment group maintained significantly better growth than those treated with Fp alone.

Yield assessment showed that Fp infection reduced the thousand-kernel weight (TKW) by 7.79%. In contrast, TB increased TKW by 12.74% compared to CK, and by 13.47% relative to the Fp group, indicating that TB helps maintain normal grain development under pathogen pressure (**Figure 2K**).

### 3.3 Regulation of antioxidant and soil enzyme activities by TB

**Figure 3:**
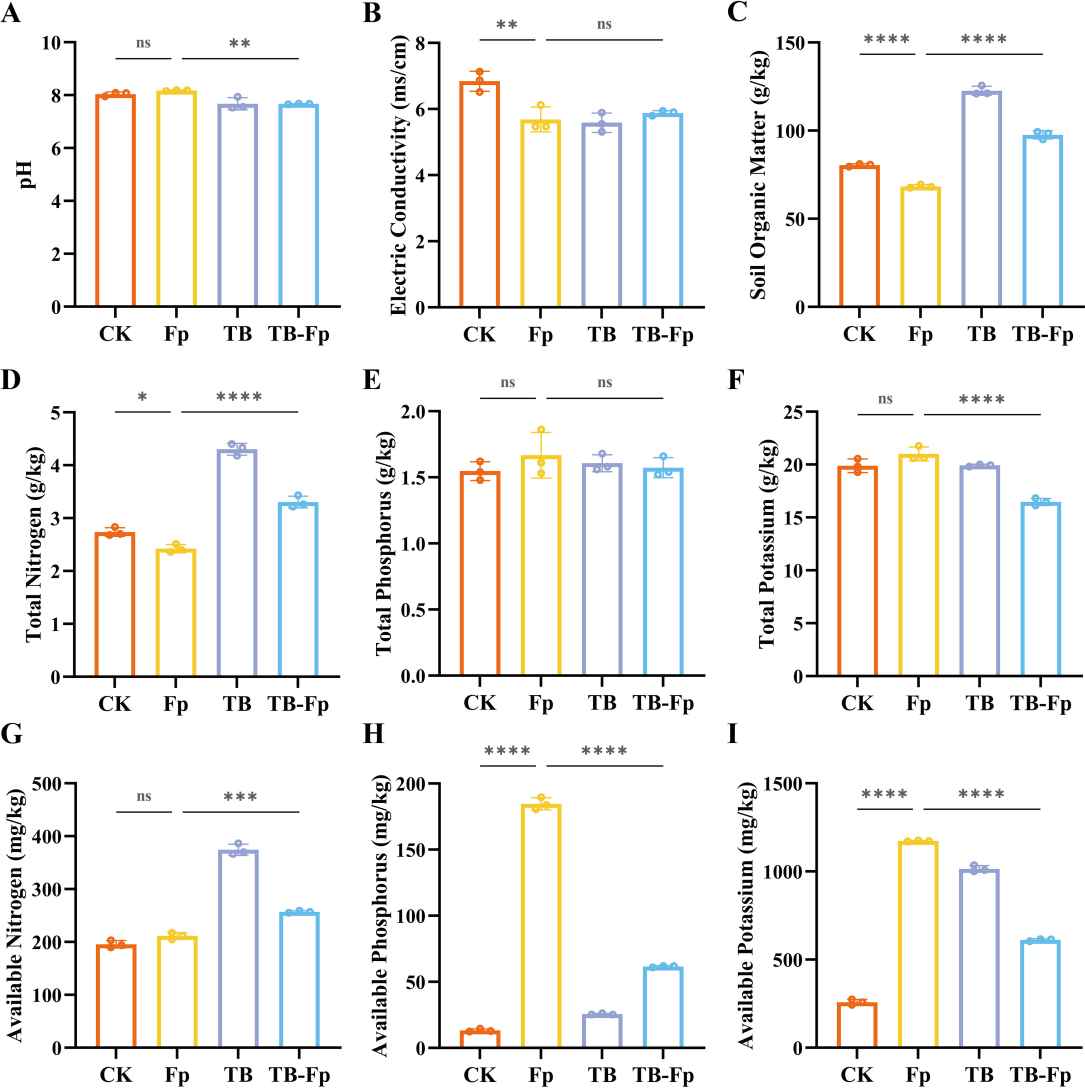
Effects of different treatments on antioxidant enzyme activities in wheat and soil enzyme activities in the rhizosphere. **(A)** Catalase (CAT); **(B)** Superoxide radicals (OFR); **(C)** Superoxide dismutase (SOD); **(D)** Peroxidase (POD); **(E)** Malondialdehyde (MDA); **(F)** Soil sucrase; **(G)** Soil acid phosphatase; **(H)** Soil urease; **(I)** Soil catalase. Significance levels: ns, not significant; *P < 0.05, **P < 0.01, ****P < 0.0001.

As shown in **Figures 3A–E**, TB-Fp treatment significantly increased catalase (CAT) activity by 53.67% compared to CK and by 88.88% compared to Fp. Fp infection markedly elevated levels of superoxide radicals (OFR) and malondialdehyde (MDA) by 234.23% and 157.28%, respectively. However, TB-Fp significantly reduced OFR and MDA by 65.08% and 66.67%, respectively, with the most substantial decrease observed for MDA. Superoxide dismutase (SOD) activity increased across all treatments, with the highest activity recorded in the TB group; TB-Fp also maintained high SOD levels under Fp stress. While Fp induced a slight increase in peroxidase (POD) activity, the difference was not statistically significant. In contrast, POD activity was significantly lower in the TB-Fp group than in the Fp group (P < 0.05).

Soil enzyme assays (**Figures 3F–L**) demonstrated that Fp infection significantly suppressed the activities of sucrase, acid phosphatase, urease, and soil CAT. In contrast, TB and TB-Fp treatments markedly enhanced these enzyme activities, indicating that TB improves the rhizosphere microenvironment and supports plant health under both normal and stress conditions.

### 3.4 TB improves rhizosphere soil physicochemical properties and nutrient availability

**Figure 4:**
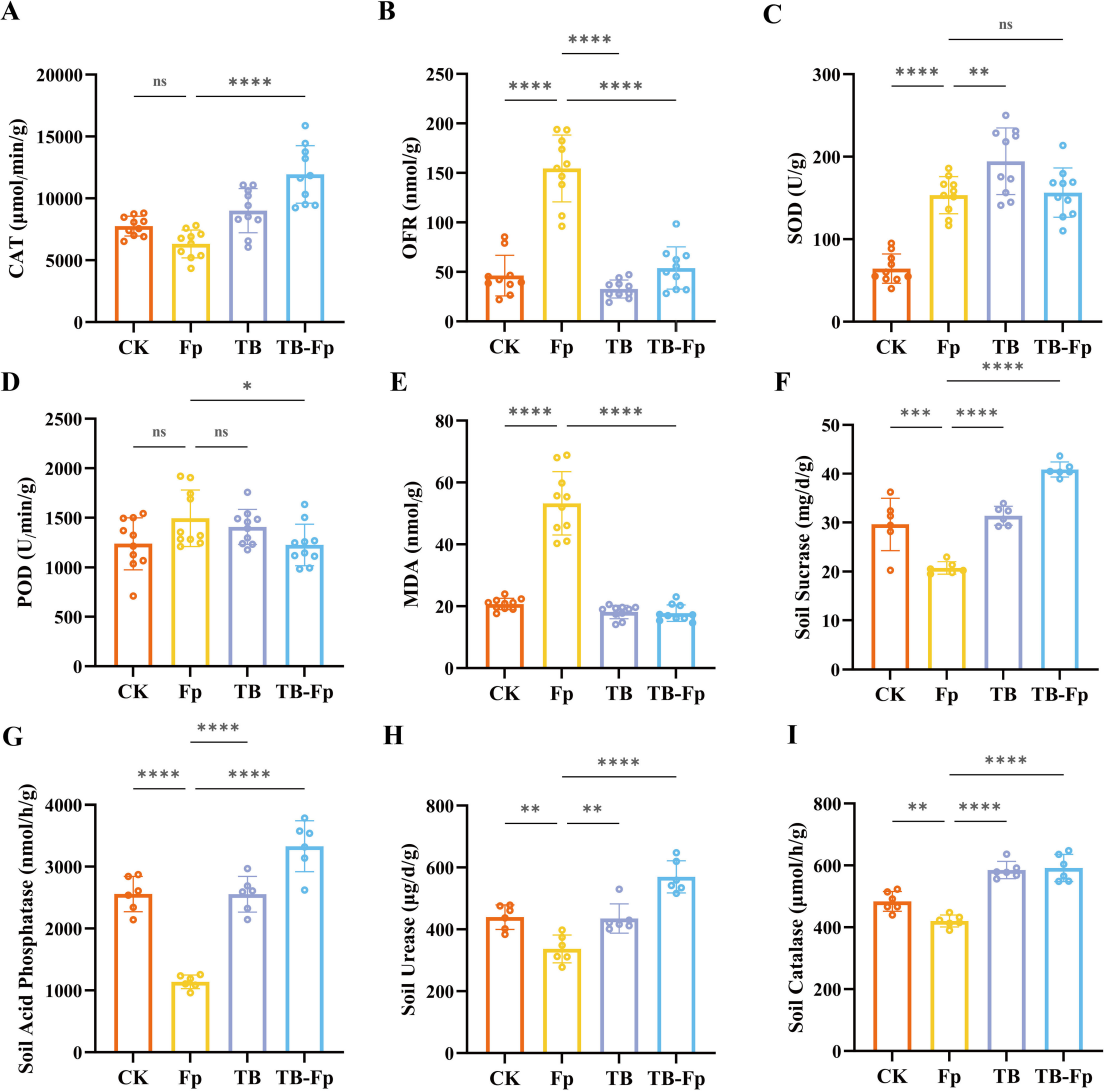
Effects of different treatments on physicochemical properties and nutrient contents of wheat rhizosphere soil. **(A)** pH; **(B)** Electric conductivity (EC); **(C)** Soil organic matter (SOM); **(D)** Total nitrogen (TN); **(E)** Total phosphorus (TP); **(F)** Total potassium (TK); **(G)** Available nitrogen (AN); **(H)** Available phosphorus (AP); **(I)** Available potassium (AK). Significance levels: ns, not significant; *P < 0.05, **P < 0.01, ****P < 0.0001.

As illustrated in **Figure 4**, Fp treatment did not significantly alter soil pH but notably reduced electrical conductivity (EC), organic matter (OM) content, and total nitrogen (TN). In contrast, TB treatment significantly increased both OM and TN levels, while TB-Fp partially restored the losses caused by Fp infection. Regarding nutrient availability, TB significantly enhanced available nitrogen content. Interestingly, Fp treatment was associated with elevated levels of available phosphorus and potassium, which may reflect disease-related tissue damage and altered nutrient turnover under infection (Tong et al., 2025; Steffens and Rasmussen, 2016).

### 3.5 SynComs significantly altered the microbial diversity and community composition in the rhizosphere soil

**Figure 5:**
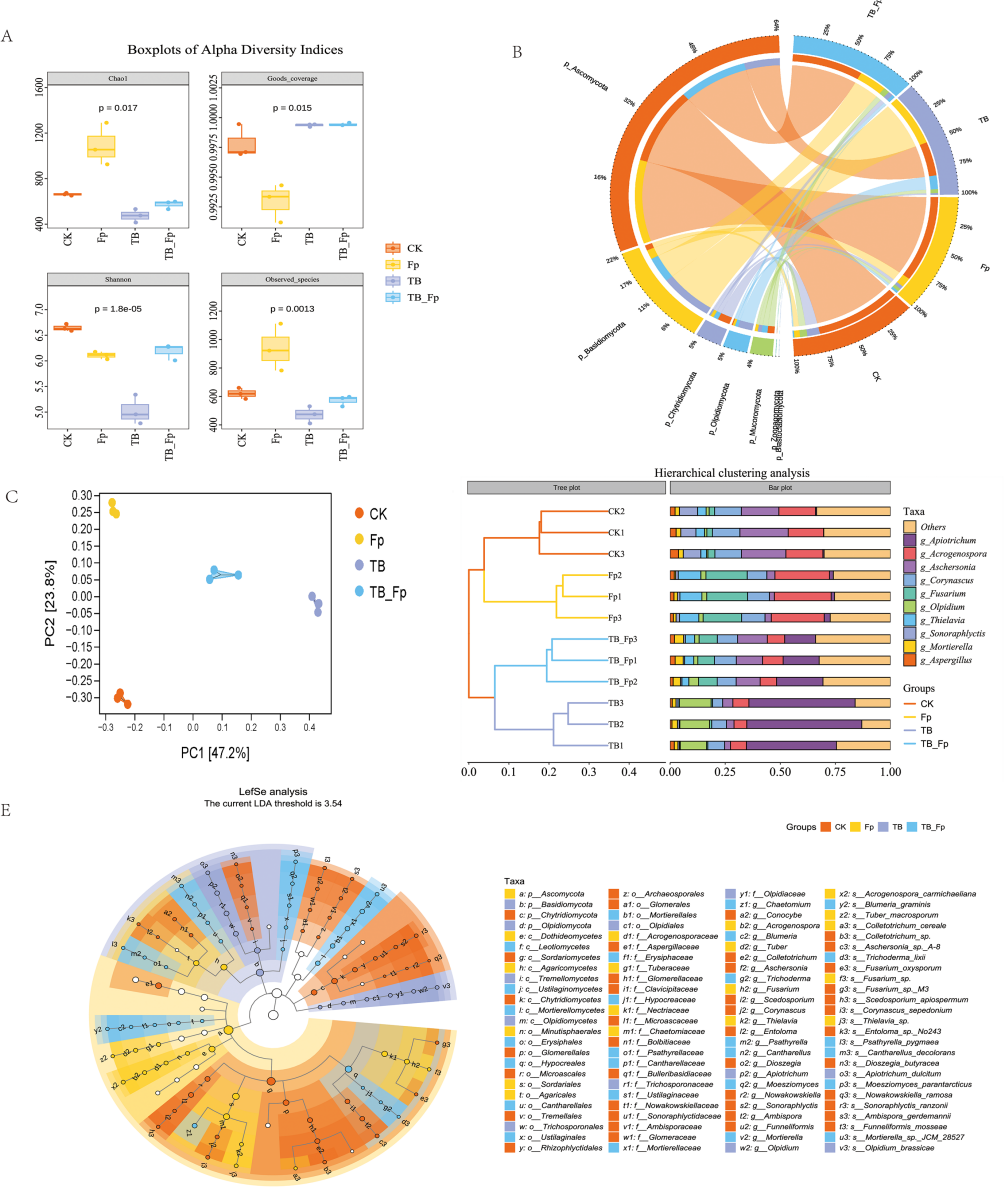
Rhizosphere microbial diversity and community composition under different treatments. **(A)** Boxplots of α-diversity indices. **(B)** Circos plots of dominant Fungi. **(C)** Principal coordinate analysis (PCoA) of microbial communities. **(D)** Hierarchical clustering and taxonomic distribution of Fungi. **(E)** LEfSe analysis of Fungi.

Through PacBio sequencing, a total of 47,635 bacterial and 8,007 fungal sequences were identified. Circos plots were generated using the online tool (https://www.genescloud.cn/chart/InterSpeciesCircos) to illustrate the composition and abundance of bacteria and fungi (**Figure 5D**) at the phylum level. Specifically, 35 bacterial phyla were identified, which were further divided into four groups across all samples, including 79 classes, 167 orders, 368 families, 944 genera, and 2,624 species. For fungi, 7 phyla were determined, which included 33 classes, 90 orders, 208 families, 410 genera, and 798 species. Additionally, the alpha diversity analysis revealed significant changes in the microbial community diversity under different treatments. Alpha diversity differed among treatments. To avoid redundancy, we focus on Chao1 (estimated richness) and Shannon (richness and evenness), which capture complementary aspects of within-sample diversity (**Figure 5B**). Across treatments, these indices showed treatment-dependent changes (P < 0.05 for selected comparisons), whereas not all alpha-diversity metrics exhibited uniform trends. Overall, these results indicate that application of the synthetic community modulated rhizosphere microbial diversity and community structure under the tested conditions, rather than uniformly increasing diversity across all treatments.

### 3.6 SynComs altered the structure of the microbial community in the rhizosphere soil as well as the composition of core species

Based on Bray–Curtis dissimilarity (relative abundance-based), hierarchical clustering grouped samples into distinct clusters (**Figure 5F**), indicating clear treatment-associated shifts in rhizosphere community structure. The hierarchical clustering analysis revealed significant differences in bacterial community composition among the different treatment groups (CK, Fp, TB, TB-Fp). The Fp group exhibited changes in certain microbial populations, especially an increase in *Pseudomonas* and *Burkholderia*. The TB treatment was associated with changes in multiple taxa, including an increase in *Rhizobium*. Notably, several ASVs annotated as *Xanthomonas* also increased in some treatments; however, because *Xanthomonas* includes both pathogenic and non-pathogenic lineages, we do not infer beneficial functions based on genus-level assignments alone. Additionally, significant differences were observed in the fungal community composition. The CK group’s microbial community was dominated by *Aspergillus* and *Corynascus*, while the Fp group (diseased group) exhibited an increase in pathogenic fungi such as *Fusarium* and *Thielavia*. The TB group displayed stronger microbial diversity, especially with an increased abundance of beneficial fungi such as *Mortierella* and *Sonoraphyctis*, indicating the growth-promoting effects of SynComs. The TB-Fp group showed the most significant changes in microbial community composition, especially in suppressing pathogenic *Fusarium* while increasing the abundance of beneficial fungi like *Mortierella* and *Thielavia*. This demonstrates that SynCom disease-control measures have a significant role in suppressing diseases and improving the microbial community structure, positively affecting soil microbial community composition (**Figure 5G**).

Through LefSe analysis, we observed significant differences in the LDA (Linear Discriminant Analysis) scores of microbial communities across different treatment groups (CK, Fp, TB, TB-Fp), indicating notable changes in the microbial composition between these groups. Specifically, the Fp group (diseased group) exhibited an increased abundance of pathogenic microorganisms, particularly Aspergillus and Corynascus. The increase in these pathogenic fungi is likely associated with the occurrence of plant diseases, suggesting that the disease has negatively impacted the soil microbial community, leading to an elevation in the abundance of pathogenic microbes. In contrast, the TB group (SynCom growth-promoting group) showed stronger microbial diversity, especially with an increase in beneficial microorganisms like *Mortierella* and *Sonoraphyctis*. This indicates that the introduction of SynComs effectively promoted plant growth and improved the microbial structure of the rhizosphere soil. *Mortierella* and *Sonoraphyctis* are known beneficial microbes that play important roles in soil health. They help plants absorb more nutrients, improve soil structure, and inhibit the growth of pathogenic microorganisms, thus creating a healthier environment for plant growth. Further analysis showed that the TB-Fp group (SynCom disease-control group) exhibited the most significant changes in microbial community composition. In this group, the abundance of *Fusarium* decreased, while the abundance of beneficial microbes such as *Mortierella* and *Thielavia* increased. *Fusarium* is a key pathogen responsible for WCR and other diseases. Its reduced abundance indicates the successful suppression of pathogenic fungi by the minimal SynCom. Additionally, the increased presence of *Thielavia* and *Mortierella* further supports the idea that SynComs play a vital role in restoring soil health and promoting plant growth. These beneficial microbes not only enhance nutrient uptake in plants but also improve soil organic matter content, boost plant immunity, and help plants better withstand environmental stresses and diseases (**Figure 5H**).

### 3.7 Microbial co-occurrence network analysis

To elucidate the structural responses of microbial communities to *Fusarium pseudograminearum* (Fp) infection and minimal SynCom inoculation, fungal and bacterial co-occurrence networks were constructed for four treatments: CK, Fp, TB, and TB-Fp. The structural characteristics and robustness of rhizosphere microbial networks differed significantly among treatments. Under pathogen stress induced by Fp, the fungal co-occurrence network showed reduced connectivity, a lower average node degree (K = 1.83), and decreased modularity (0.348), indicating a breakdown of cooperative structures and impaired ecological integration within the fungal community. The network diameter reached its highest value in the Fp group (7.9), reflecting reduced communication efficiency and an increased risk of functional fragmentation.

**Figure 6:**
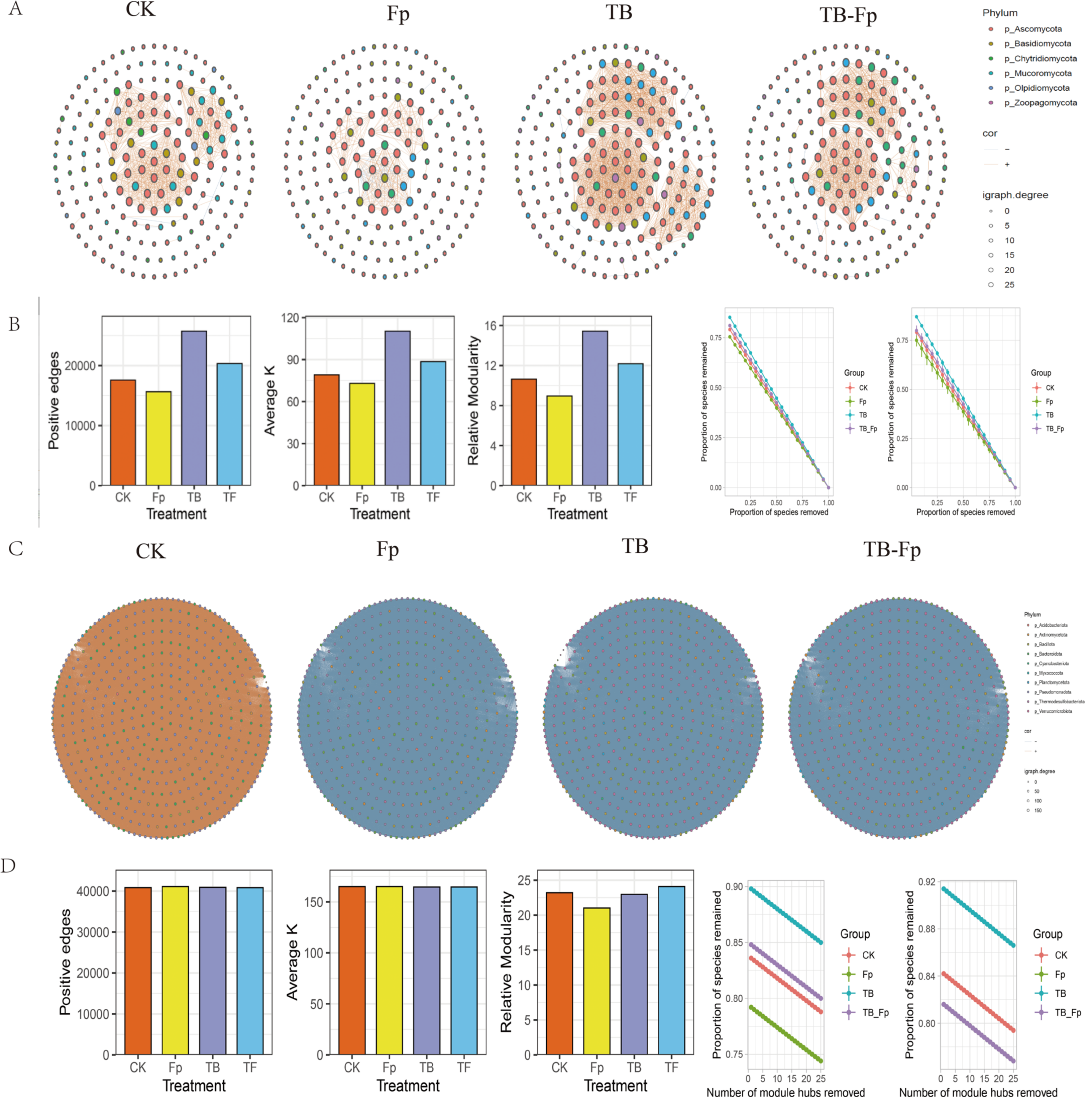
Co-occurrence network structures of rhizosphere microbial communities under different treatments. **(A)** Network visualizations of fungal taxa for each treatment (CK, Fp, TB, TB-Fp), with node colors indicating phylum and size representing degree. **(B)** Bar charts showing positive edges, average connectivity **(k)**, and relative modularity for each treatment. **(C)** Network visualizations after module hub removal. **(D)** Bar charts summarizing network properties and species removal proportion after module hub removal across treatments.

Application of the minimal SynCom (TB) increased the number of positive associations in the fungal network (edges = 194), elevated the average node degree (K = 3.14), and improved modularity (0.426) (**Figures 6A–C**). In the TB-Fp group, these topological enhancements were largely retained despite the presence of the pathogen (edges = 185, K = 2.97, modularity = 0.419), suggesting that the introduced microbial consortium buffered structural disruption. Compared with Fp alone, the TB-Fp network exhibited more densely connected hubs and shorter average path lengths, features characteristic of resilient microbial systems (**Figure 6**).

Robustness analysis indicated that microbial networks under TB and TB-Fp treatments maintained higher natural connectivity under both random (**Figure 6H**) and targeted node removal. In fungal networks, natural connectivity values under random node loss were 0.126 (TB) and 0.123 (TB-Fp), while the Fp group decreased to 0.096. Hub-targeted simulations showed a similar trend, with slower decay in TB and TB-Fp compared to Fp. Interestingly, these structural responses were more pronounced in fungal communities, while bacterial networks showed less variation across treatments (**Supplementary Figure S1**).

Neutral community model (NCM) analysis revealed that bacterial communities were largely governed by stochastic processes across all treatments, with consistently high model fits (R^2^ ≈ 0.60–0.63; overall R^2^ = 0.738) and ~100% of taxa fell within the neutral model prediction. This indicates that bacterial assembly was stable and only marginally affected by pathogen or minimal SynCom inoculation. In contrast, fungal communities exhibited weaker neutral signals (overall R² = 0.673) and greater treatment-dependent variation: pathogen inoculation (Fp) markedly reduced model fit (R^2^ = 0.452), whereas SynCom treatment increased it to the highest level (R^2^ = 0.613), with TB-Fp also higher than Fp (R^2^ = 0.578). These findings suggest that the minimal SynCom had little effect on bacterial assembly but substantially mitigated deterministic selection in fungi, enhancing their stochastic assembly under pathogen pressure (**Supplementary Figure S2**).

### 3.8 TB treatment altered and enhanced the composition of rhizosphere soil metabolites, leading to the accumulation of growth-promoting and stress-responsive compounds

**Figure 7:**
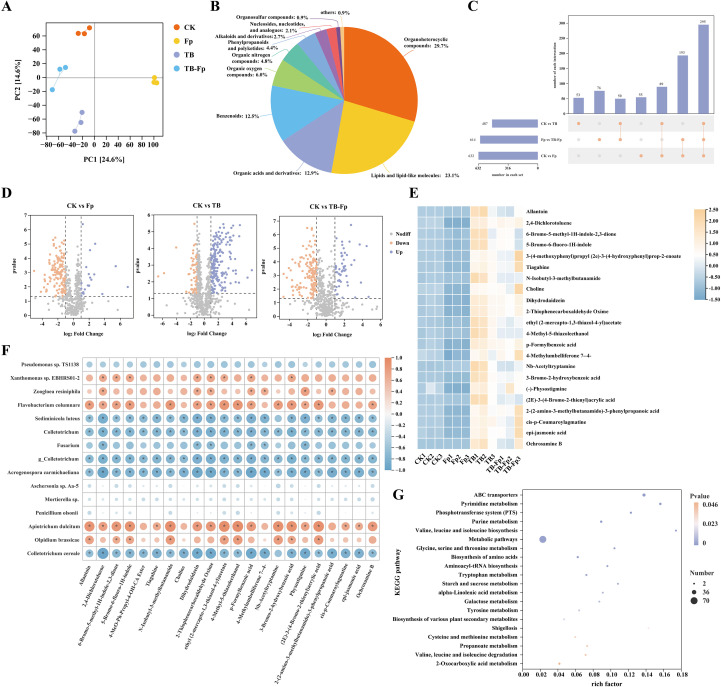
Metabolomic analysis of rhizosphere soil under different treatments. **(A)** Principal component analysis (PCA) of soil metabolite profiles. **(B)** Classification of detected metabolites. **(C)** UpSet plot of unique and shared metabolites. **(D)** Volcano plots of differential metabolites between CK vs. Fp, CK vs. TB, and CK vs. TB-Fp. **(E)** Heatmap of selected differential metabolites. **(F)** Correlation heatmap between representative metabolites and microbial taxa. **(G)** KEGG pathway enrichment analysis of differential metabolites. *P < 0.05.

Untargeted metabolomic analysis revealed significant alterations in rhizosphere soil metabolite composition across different treatments. Principal component analysis (PCA) showed clear separation among the four groups, with PC1 and PC2 explaining a combined 37.9% of total variance. CK and Fp samples clustered together, while TB and TB-Fp formed a distinct cluster, indicating that both pathogen pressure and microbial inoculation significantly affected the rhizosphere metabolic landscape (**Figure 7A**).

A total of 1,338 metabolites were detected and annotated. According to KEGG superclass classification, lipids and lipid-like molecules accounted for the largest proportion (46.3%), followed by organic oxygen compounds (14.9%), phenylpropanoids and polyketides (13.2%), organic acids (8.1%), and benzenoids (7.4%) (**Figure 7B**). Upset analysis showed that 65 metabolites were uniquely enriched in the TB-Fp group and 42 in the TB group, suggesting that the minimal SynCom induced specific metabolic responses under pathogen challenge (**Figure 7C**).

Trend clustering analysis partitioned the differential metabolites into ten distinct groups. Cluster 2 (31 metabolites) exhibited a pronounced enrichment under the TB-Fp treatment, showing a clear upregulation trajectory in response to the combined action of minimal SynCom inoculation and pathogen stress. Metabolites within this cluster were predominantly organic acids, amino acid derivatives, and signaling compounds, including dihydrodaidzein, cis-p-coumaroylagmatine, and choline. These compounds are closely associated with osmotic adjustment, energy metabolism, and defense signaling, suggesting their central role in maintaining metabolic homeostasis under pathogen challenge. Furthermore, correlation analyses revealed significant linkages between Cluster 2 metabolites and multiple microbial taxa, underscoring their pivotal contribution to the microbiome-mediated metabolic response during pathogen pressure (**Figure 7F**).

### 3.9 Integrative analysis reveals nutrient–enzyme–microbiome pathways underpinning TB-induced disease resistance and yield benefits

KEGG pathway enrichment analysis indicated that TB and TB-Fp significantly enriched multiple metabolic pathways, including ABC transporters, branched-chain amino acid biosynthesis (valine, leucine, and isoleucine), glutathione metabolism, glycerophospholipid metabolism, and nucleotide sugar metabolism (**Figure 7G**). Notably, the TB-Fp treatment was associated with enrichment of a broader set of pathways, including modules related to secondary metabolite biosynthesis and xenobiotic degradation.

**Figure 8:**
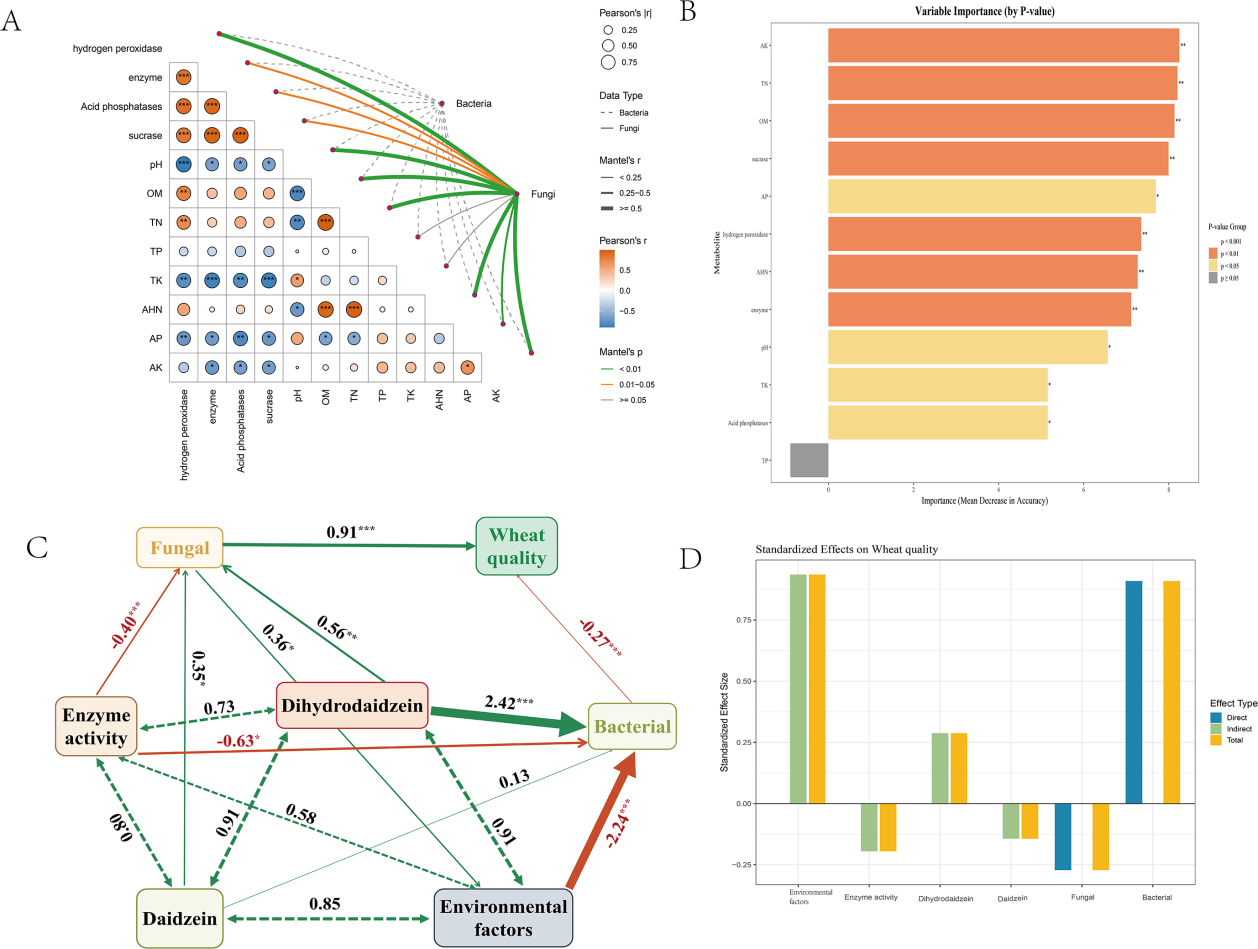
Integrative analysis of soil–enzyme–microbiome pathways affecting wheat quality. **(A)** Mantel test showing correlations between soil properties, enzyme activities, and microbial communities. **(B)** Random forest analysis of variable importance. **(C)** Structural equation model (SEM) of environmental factors, enzyme activity, metabolites, microbial communities, and wheat quality. **(D)** Standardized direct, indirect, and total effects of different factors on wheat quality. *P < 0.05, **P < 0.01, ****P < 0.0001.

The Mantel test results revealed significant correlations between soil physicochemical factors and both rhizosphere bacterial and fungal communities. In particular, variables such as total phosphorus (TP), total potassium (TK), available phosphorus (AP), available hydrolyzable nitrogen (AHN), and acid phosphatase activity exhibited strong associations with microbial community structures (Mantel’s r > 0.5, P < 0.05), suggesting their pivotal roles in shaping the rhizosphere microbiome (**Figure 8A**). Complementary random forest analysis further identified TP, acid phosphatase, TK, and pH as the most influential predictors of microbial community variation. Among them, TP and acid phosphatase showed significantly higher importance scores (P < 0.001), underscoring the critical influence of soil nutrient status and enzymatic activity on microbial assemblages (**Figure 8B**).

Building upon these findings, the results of the structural equation model demonstrate that the minimal SynCom TB significantly enhanced wheat quality by systematically regulating soil environmental conditions, enzyme activity, and the structure of rhizosphere microbial communities. The model exhibited a good fit, explaining 78.0%, 90.7%, and 94.8% of the variance in the fungal community, bacterial community, and wheat quality, respectively. Specifically, soil environmental factors had a strong negative effect on the fungal community and a positive influence on the bacterial community, while enzyme activity suppressed fungal abundance. In contrast, daidzein markedly promoted the fungal community, suggesting that plant-derived metabolites contribute to the assembly of beneficial fungi. Although dihydrodaidzein did not show significant effects in the current model, modification indices indicated its potential positive role in improving wheat quality. Among the microbial components, the bacterial community emerged as the primary driver with a strong positive impact on wheat quality, whereas the fungal community exhibited a significant negative effect, likely due to the presence of pathogenic taxa. This provides a mechanistic foundation for the application of SynComs in sustainable agriculture (**Figures 8C, D**).

## 4 Discussion

### 4.1 A minimal cross-kingdom SynCom confers growth promotion and WCR suppression

**Figure 9:**
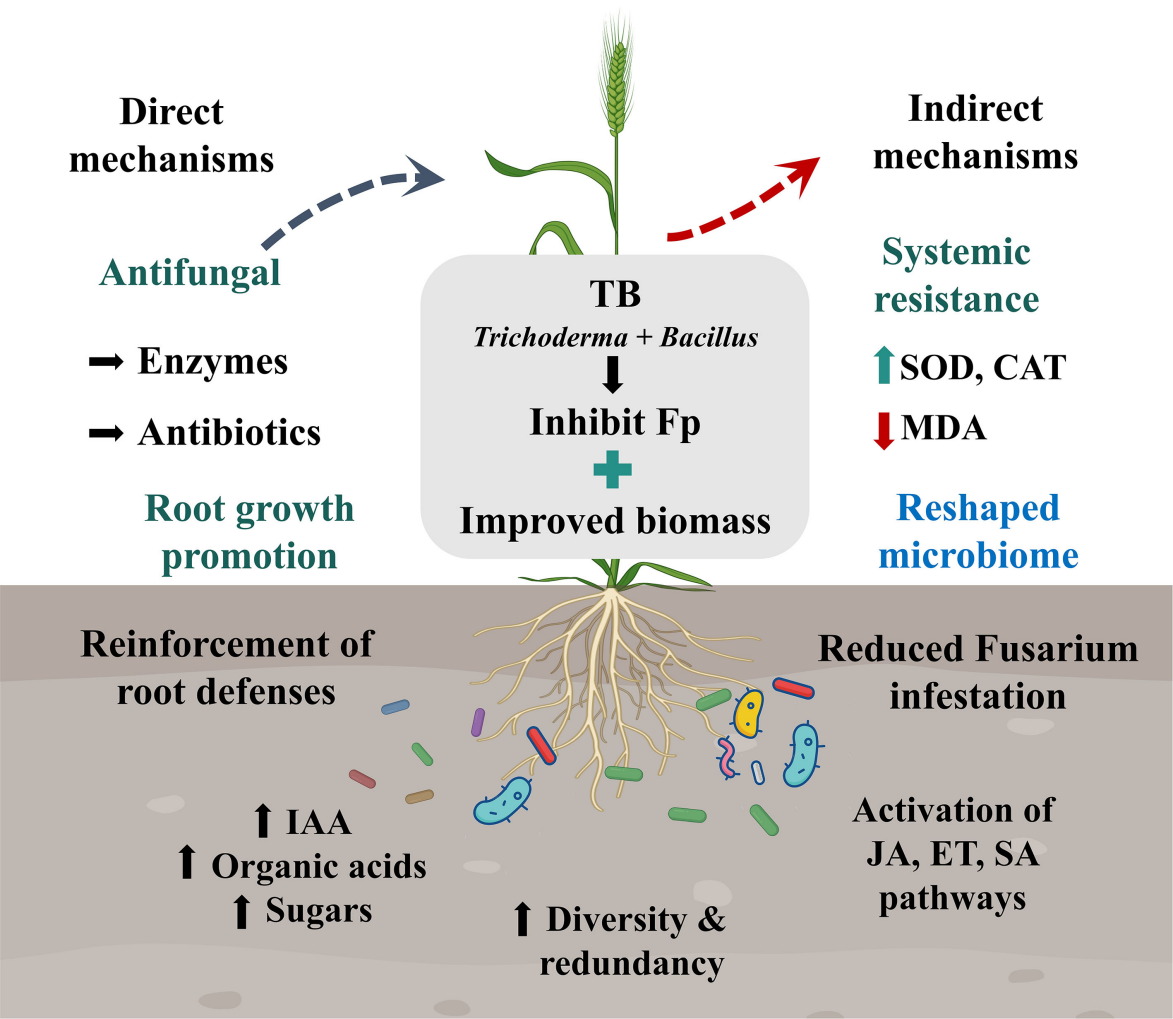
Proposed mechanistic model of the synthetic microbial consortium (TB) in wheat.

Our findings demonstrate that a tailored minimal SynCom can substantially suppress *Fusarium*-induced crown rot in wheat while bolstering plant growth and health. This protective outcome is not attributable to a single mechanism but rather emerges from an integrated plant–microbiome interaction. In essence, wheat plants under pathogen attack appear to “recruit” a beneficial rhizosphere consortium through specific root exudate signals, and the introduced SynCom amplifies this effect by restructuring the microbiome into a disease-suppressive community. The result is a fortified rhizosphere environment that functions as an extension of the plant’s immune system, providing both direct antagonism of the pathogen and enhanced host resilience (**Figure 9**).

### 4.2 Rhizosphere metabolic reprogramming under pathogen pressure and SynCom treatment

A potential element underlying disease suppression is the capacity of plants to reshape rhizosphere chemistry under stress, a concept often discussed in the context of the “cry-for-help” framework. In our study, metabolomics was performed on rhizosphere soil extracts rather than on collected root exudates; therefore, the detected enrichment of specific compounds should be interpreted as rhizosphere-level metabolic shifts that may arise from a combination of plant release, microbial metabolism/biotransformation, and pathogen-driven turnover. Nevertheless, pathogen recognition is known to be associated with broad changes in rhizosphere chemistry, and stress-altered exudation patterns have been reported to selectively enrich protective members of the soil microbiome (Liu et al., 2024b). For example, heat-stressed plants can secrete purine and pyrimidine nucleotides that function as foraging cues, promoting beneficial rhizobacteria and fungi while constraining root-rot pathogens. Consistent with these observations, we detected enrichment of several metabolites—particularly flavonoid-related compounds—in the rhizosphere under *Fusarium* challenge and SynCom treatment. Flavonoids are well-established mediators of plant–microbe interactions and can influence microbial chemotaxis, colonization, and activity (Wang et al., 2022a). Moreover, under biotic or abiotic stress, plants frequently show increased accumulation and/or release of phenolics and flavonoids, which may function both as antimicrobial metabolites and as ecological cues shaping microbial community assembly (Wu et al., 2025). In our dataset, daidzein abundance was positively correlated with the relative abundance of *Mortierella*, a genus often associated with plant-beneficial functions. However, given that rhizosphere metabolites are not exclusively plant-derived, this association does not demonstrate a strictly plant-exudate origin of daidzein or a direct recruitment mechanism. Instead, the observed pattern is consistent with several non-mutually exclusive possibilities: (i) pathogen- and/or SynCom-induced changes in root metabolism and release, (ii) microbial production or biotransformation of flavonoid-like metabolites, and/or (iii) altered turnover and retention of these compounds in the rhizosphere. Taken together, our results support rhizosphere metabolic reprogramming under pathogen pressure and SynCom treatment, and they are compatible with (but do not by themselves prove) a “cry-for-help”-type process. We therefore restrict our interpretation to the observed associations at the rhizosphere level and do not assign metabolite origin or recruitment causality based on the present dataset.

### 4.3 Functional complementarity and synergy of the two-member SynCom

Importantly, the introduced SynCom greatly enhanced the efficacy and consistency of this recruitment process, leading to a profound shift in rhizosphere community structure and function. The two-member SynCom we employed (comprising *T. harzianum* T19 and *B. rugosus* PM16) was designed to provide complementary benefits, and our results confirm a strong synergistic interaction. Cross-kingdom consortia of beneficial microbes (fungi + bacteria) have been shown to outperform single-strain inoculants in suppressing diseases (Zhou et al., 2022a), and we observed similarly that our fungal–bacterial SynCom achieved ~71% biocontrol efficacy against WCR while significantly promoting wheat growth. This synergy is underpinned by the multi-faceted roles each member plays: *Trichoderma* T19 directly antagonizes the pathogen through mycoparasitism and antifungal metabolite production, whereas *Bacillus* PM16 contributes by producing growth hormones (IAA), solubilizing nutrients, and likely secreting antibiotics and siderophores that inhibit pathogens. By combining these functions, SynCom provided both robust pathogen suppression and growth promotion simultaneously. Moreover, the two strains were compatible and exhibited no antagonism towards each other, enabling them to cooperate in colonizing the wheat root niche as a cohesive unit. This cooperative behavior is crucial, as it allows the SynCom to act as a keystone assemblage that reorganizes the surrounding microbial community in favor of plant health.

### 4.4 Microbiome remodeling and network stabilization under pathogen pressure

Following SynCom inoculation, the rhizosphere shift we observed can be interpreted as an accelerated transition toward a disease-suppressive state, in which community-level properties collectively constrain pathogen establishment rather than relying on a single antagonist (Mendes et al., 2013; Mazzola and Freilich, 2017; Mendes et al., 2011). Mechanistically, this outcome is consistent with layered ecological processes. First, SynCom members may generate priority effects and niche pre-emption at the root-soil interface, occupying infection courts and consuming limiting resources, thereby increasing invasion resistance in a manner that is often better explained by competitive network structure than by diversity alone (Wei et al., 2015). Second, SynCom strains can impose interference competition via antibiotics, lipopeptides, hydrolytic enzymes, and biofilm-associated exclusion—well-established modes of action in rhizosphere biocontrol that also contribute to suppressive soil phenotypes ( (Raaijmakers and Mazzola, 2012; Schlatter et al., 2017). Third, SynCom colonization may indirectly remodel the resident microbiome through host-mediated selection (i.e., altered rhizodeposition and immune activation), a mechanism demonstrated to assemble protective consortia after pathogen challenge (Berendsen et al., 2018). In support of metabolite-guided recruitment, the isoflavone daidzein has been shown to shape rhizosphere bacterial community composition *in situ*, indicating that plant specialized metabolites can act as selective cues that amplify beneficial guilds beyond simple nutrient effects (Okutani et al., 2020). In addition, enrichment of *Mortierella* is frequently linked with soil health and pathogen suppression potential in diverse cropping systems, suggesting that fungal commensals may contribute to buffering against *Fusarium* takeover (Wang et al., 2022b; Ozimek and Hanaka, 2021). Finally, the network-level stabilization we detected is consistent with reports that disease often disrupts microbial co-occurrence patterns and ecological functions, whereas higher connectivity and cooperative structure can enhance community resilience and dampen disturbance propagation—thereby indirectly sustaining nutrient cycling and other functions that support plant defense (Zhou et al., 2022a; Gao et al., 2021; Wei et al., 2018). Taken together, these lines of evidence support the view that SynCom application does not merely “add beneficial strains,” but rather reconfigures competitive and cooperative interactions in the resident microbiome, creating a rhizosphere environment that is less permissive to *Fusarium* proliferation and more conducive to stable plant-soil functioning.

### 4.5 SynCom-associated immune priming and oxidative stress mitigation

Multiple lines of evidence in our study point to the activation of plant innate immunity by the SynCom, indicating a significant contribution of microbe-mediated immune priming to disease suppression. Wheat plants treated with the SynCom showed enhanced activity of antioxidant enzymes (e.g. catalase, superoxide dismutase) and lower accumulation of oxidative damage markers during *Fusarium* infection, compared to uninoculated plants. This suggests that the plant’s defensive machinery was already elevated or more rapidly induced – a classic outcome of induced systemic resistance (ISR) or priming caused by beneficial microbes (Gámez-Arcas et al., 2021). Certain rhizobacteria and biocontrol fungi are known to elicit ISR by triggering plant immune pathways (such as jasmonic acid/ethylene signaling), leading to fortification of the plant’s cell walls, production of antimicrobial compounds, and a heightened state of alert to incoming pathogens. In our SynCom, *Bacillus* likely acted as an ISR elicitor; members of this genus possess microbe-associated molecular patterns (e.g. flagellin, lipopeptides) that plant roots recognize, resulting in systemic resistance to diverse pathogens. *Trichoderma* spp. also are well documented to induce defense genes and biochemical defenses in plants, including the accumulation of phenolics and pathogenesis-related proteins (Thapa et al., 2025). The combination of these two beneficial microbes in our SynCom may thus have provided a dual boost to the plant’s immune system, keeping it primed against *Fusarium* invasion. Notably, the disease suppression observed was far greater than what could be explained by direct pathogen antagonism alone, implying that the plant’s reinforced immunity was a key factor in limiting *Fusarium* spread. This mirrors findings from other systems where SynCom-mediated protection was linked to enhanced host immune responses. In the tomato–*Fusarium* wilt model, for instance, a cross-kingdom SynCom conferred strong disease resistance concomitant with upregulation of the plant’s defense pathways. Our results are in line with these reports and reinforce the concept that effective biocontrol in the rhizosphere invariably involves both microbial warfare and host defense fortification.

### 4.6 Chemical signaling and cross-talk as a coordinating layer

Finally, it is important to recognize that chemical signaling and cross-talk may contribute to orchestrating the above interactions. The rhizosphere is a chemically dynamic arena where plant exudates, microbial metabolites, and even pathogen-derived molecules continuously shape community behavior. Our discussion has highlighted how plant-derived signals (nucleotides, flavonoids, etc.) attract beneficial microbes and modulate their activity. Conversely, the microbes produce signals that influence the plant and each other (Gámez-Arcas et al., 2021). For example, beneficial bacteria can secrete quorum-sensing molecules or hormones that alter root exudation patterns or biofilm formation. There is evidence that plant metabolites can intercept or mimic microbial quorum signals; certain flavonoids, for instance, have been reported to interfere with bacterial quorum-sensing and virulence gene expression (Wang et al., 2025a). In this context, signaling cross-talk could plausibly occur in our SynCom system. The introduced *Bacillus* and *Trichoderma* strains may communicate chemically to coordinate colonization (supporting compatibility and cooperation), and their metabolites could be perceived by the plant (potentially engaging ISR) and/or by indigenous microbes. Microbial volatile compounds (e.g., those produced by *Bacillus*) may further influence plant growth and immunity or inhibit pathogen growth at a distance. In addition, small diffusible metabolites from the SynCom could affect *Fusarium* physiology (for example, by modulating germination or virulence-associated processes) (Gámez-Arcas et al., 2021). Importantly, these QS- and volatile-mediated effects are proposed as working hypotheses and were not directly tested in the present study. While disentangling these signaling networks was beyond the scope of this work, we suggest that such chemical dialogues may have contributed to the coordinated defensive responses observed. Overall, our results are consistent with a tri-partite interaction among the plant, SynCom members, and the pathogen, in which chemical exchanges may help shape the direction and outcome of rhizosphere interactions.

In summary, our study portrays a comprehensive mechanism of wheat disease suppression under SynCom inoculation, driven by plant–microbe–metabolite interactions. When faced with *Fusarium* attack, wheat roots release distress signals that recruit and activate beneficial microbes, and a properly formulated SynCom can amplify this natural defense strategy by remodeling the microbiome and priming the plant’s immunity. Through a combination of microbial niche competition, antibiosis, and cooperative network formation, alongside host immune priming and signal exchange, the SynCom-treated rhizosphere becomes an inhospitable territory for the pathogen. Not only do these findings advance our understanding of the “cry-for-help” paradigm in a cereal crop pathosystem, but they also underscore the translational potential of microbiome engineering in agriculture. By leveraging indigenous signaling pathways and microbial partnerships, we can design SynCom interventions that reinforce the plant’s own defenses and establish durable disease-suppressive soils. Such ecologically informed strategies hold promise for sustainable crop protection, reducing reliance on chemical pesticides and fostering resilient agroecosystems.

## 5 Conclusion

In conclusion, our study demonstrates that a targeted cross-kingdom SynCom can effectively suppress the devastating soil-borne disease WCR while concurrently promoting wheat growth and yield by reshaping the rhizosphere environment. By integrating beneficial microbes to fortify the soil microbiome, we created a disease-suppressive and growth-enhancing ecosystem, showcasing a novel and effective biocontrol approach. This proof-of-concept highlights the potential of microbiome engineering as a sustainable alternative to chemical pesticides for managing soilborne crop diseases. The insights gained into plant–microbe–pathogen interactions—through multi-omics analyses of community dynamics, network stability, and metabolite profiles—underscore the importance of holistic strategies in crop protection. Looking ahead, refining such synthetic consortia for different crops and conditions, and unraveling the molecular mechanisms of their interactions, will be crucial for translating this approach into resilient agroecosystems and improving global food security.

## Data Availability

The datasets presented in this study can be found in online repositories. The names of the repository/repositories and accession number(s) can be found below: https://www.ncbi.nlm.nih.gov/, PRJNA1320663 https://www.ncbi.nlm.nih.gov/, PRJNA1320686 https://www.ebi.ac.uk/ena, MTBLS12945.
