# Supplementary material for: A minimal cross-kingdom SynCom promotes plant growth and suppresses wheat crown rot via coordinated rhizosphere microbiome remodeling
Source: Front Plant Sci. 2026 Feb 25;17:1758273. doi: 10.3389/fpls.2026.1758273 (PMC12977338; doi:10.3389/fpls.2026.1758273)
Supplement: Supplementary file 1 [file DataSheet1.docx]

Supplementary Material

# Supplementary Figures and Tables

For more information on Supplementary Material and for details on the different file types accepted, please see [here](https://www.frontiersin.org/guidelines/author-guidelines#supplementary-material).

## Supplementary Figures


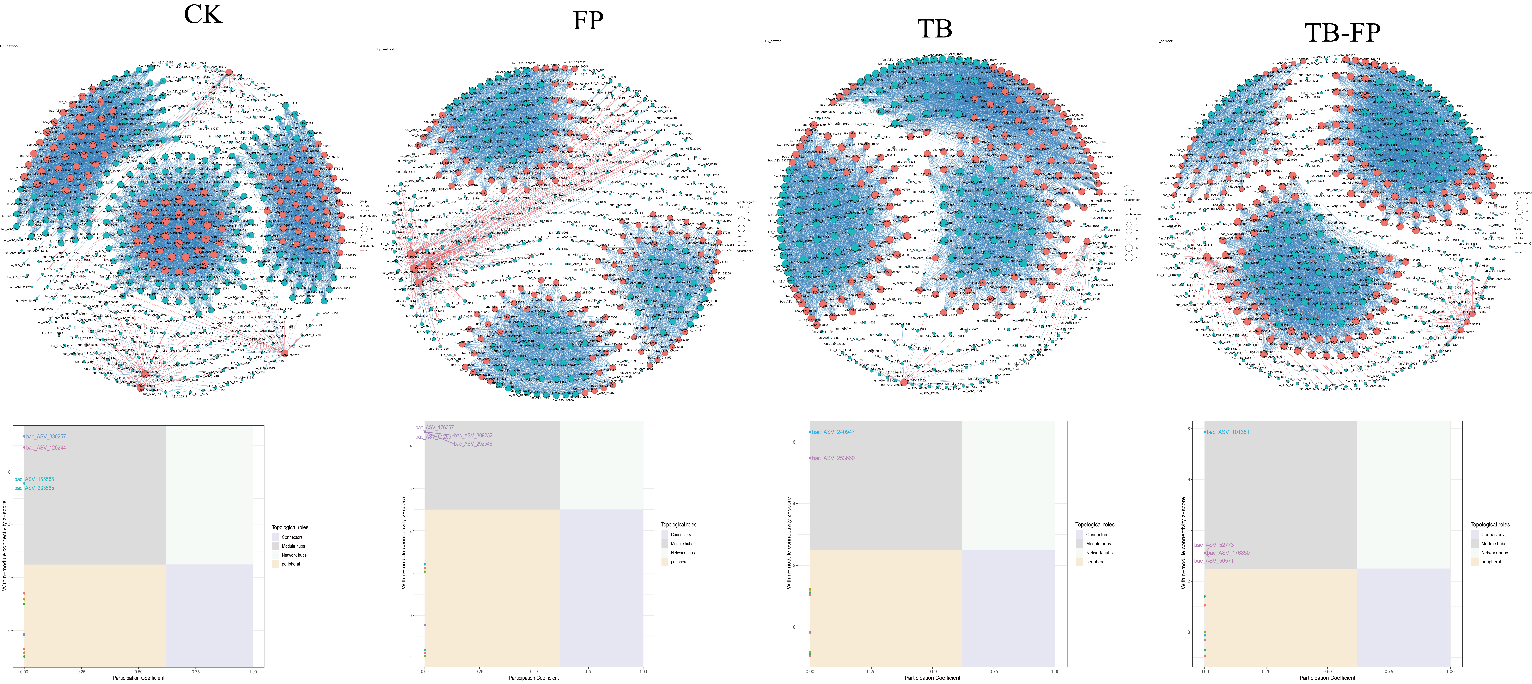
**Supplementary Figure 2.** Effects of SynCom on rhizosphere community assembly.


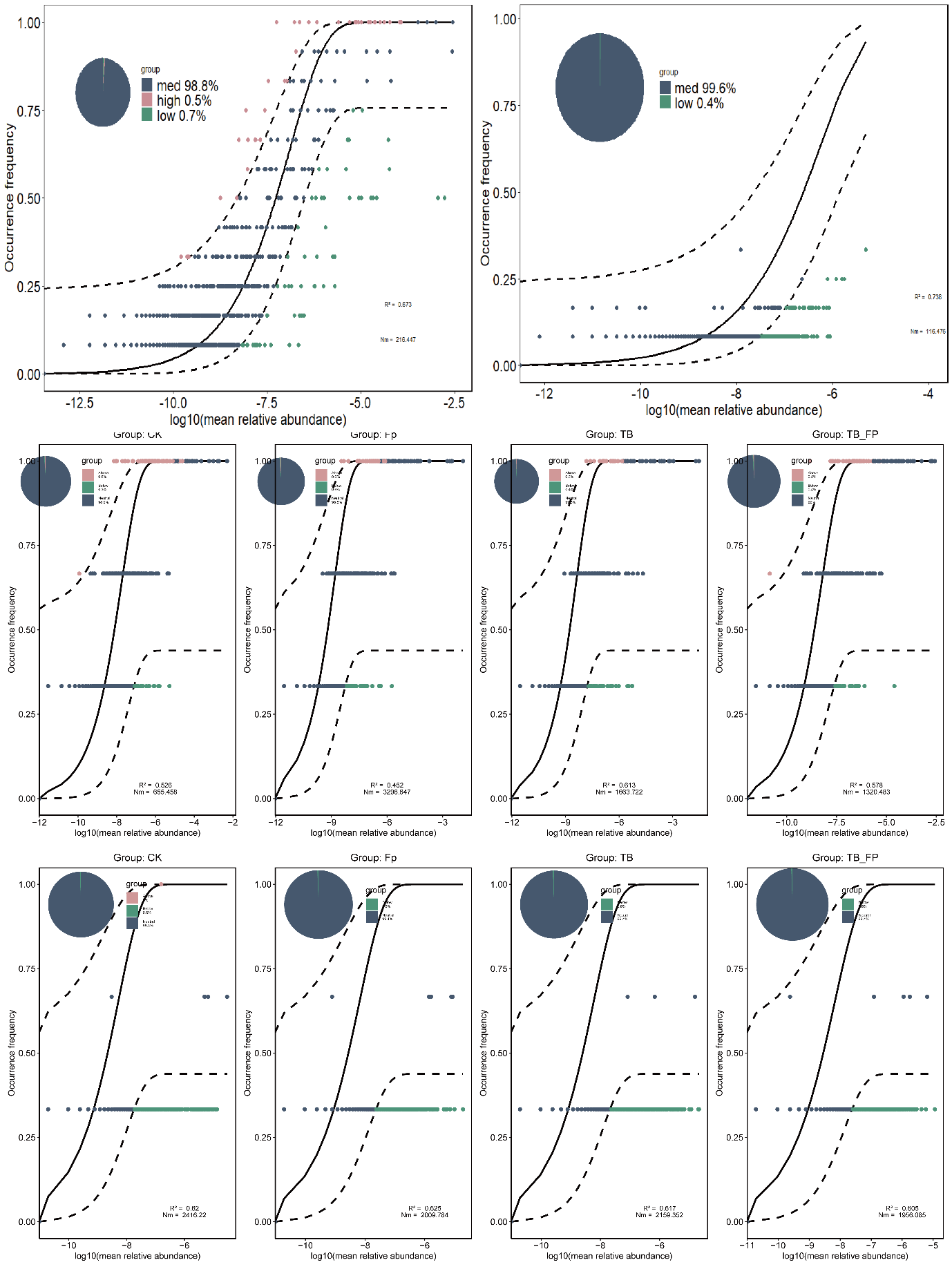


**Supplementary Figure 3.** Neutral community model (NCM) fitting of fungal and bacterial communities.

## Supplementary Tables

**Table S1** Estimated baseline physicochemical properties of bulk soil before pot establishment

| **Strain** | **pH** | **Organic matter (g/kg)** | **Electrical conductivity (mS/cm)** |
| --- | --- | --- | --- |
| **CK** | 8.09 ± 0.03 | 82.29 ± 0.85 | 7.47 ± 0.24 |

**Table S2** Strains with key plant-growth-promoting traits

| **Strain** | **Phosphate-solubilization halo diameter (cm)** | **IAA production (mg/L)** | **Siderophore halo diameter (mm)** |
| --- | --- | --- | --- |
| **PM5** | 1.27 ± 0.03 | 35.43 ± 0.40 | 14.5 ± 0.5 |
| **PM16** | 1.95 ± 0.05 | 48.67 ± 1.15 | 26.17 ± 1.04 |
| **PM13** | 1.65 ± 0.09 | 30.67 ± 1.15 | 22.67 ± 0.29 |
| **PM17** | 1.05 ± 0.05 | 16.33 ± 1.15 | 17.17 ± 0.29 |
| **PM21** | 1.10 ± 0.10 | 16.00 ± 1.00 | 17.0 ± 0.0 |
| **PM36** | 1.12 ± 0.08 | 6.59 ± 0.52 | 15.17 ± 1.26 |
| **PM37** | 1.00 ± 0.10 | 3.47 ± 0.50 | 14.67 ± 1.15 |
| **PM44** | 1.08 ± 0.02 | 13.95 ± 1.69 | 16.67 ± 0.29 |
| **PM59** | 1.15 ± 0.05 | 12.83 ± 0.76 | 16.33 ± 0.58 |
| **PM63** | 1.18 ± 0.08 | 1.16 ± 0.30 | 11.83 ± 0.76 |
| **PM76** | 1.13 ± 0.03 | 6.50 ± 0.50 | 15.17 ± 0.29 |
| **PM92** | 1.05 ± 0.00 | 18.95 ± 1.08 | 16.83 ± 0.76 |
| **PM95** | 1.08 ± 0.03 | 28.81 ± 0.17 | 17.83 ± 1.04 |
| **PM99** | 1.22 ± 0.03 | 21.36 ± 0.31 | 18.33 ± 0.58 |
